# EPR Spectra of Sintered Cd_1−x_Cr_x_Te Powdered Crystals with Various Cr Content

**DOI:** 10.3390/ma14133449

**Published:** 2021-06-22

**Authors:** Ireneusz Stefaniuk, Werner Obermayr, Volodymyr D. Popovych, Bogumił Cieniek, Iwona Rogalska

**Affiliations:** 1 College of Natural Sciences, University of Rzeszow, Rejtana 16a, 35-310 Rzeszow, Poland; bcieniek@ur.edu.pl (B.C.); iwrogalska@ur.edu.pl (I.R.); 2 Institute for Electronic Engineering, FH Joanneum, Alte Poststraße 147, 8020 Graz, Austria; werner.obermayr@fh-joanneum.at; 3 Department of Machine Science and Fundamental Technologies, Ivan Franko Drogobych State Pedagogical University, Ivan Franko Str. 24, 82100 Drogobych, Ukraine; v.popovych@dspu.edu.ua

**Keywords:** diluted magnetic semiconductors, CdTe:Cr, ferromagnetic materials, electron paramagnetic resonance, Curie temperature

## Abstract

In this paper, we show a simple method of producing ferromagnetic materials with a Curie temperature above room temperature. The electron paramagnetic resonance (EPR) spectra of Cd_1−x_Cr_x_Te (0.002 < x < 0.08) were measured with a dependence on temperature (82 K < T < 381 K). Obtained EPR lines were fitted to a Lorentz-shaped curve. The temperature dependencies of the parameters of the EPR lines, such as the peak-to-peak linewidth (H_pp_), the intensity (A), as well as the resonance field (H_r_), were studied. Ferromagnetism was noticed in samples at high temperatures (near room temperature). For a sample with a nominal concentration of chrome of x = 0.05, a very strong intrinsic magnetic field is observed. The value of the effective gyromagnetic factor for this sample is g_e_ = 30 at T = 240 K. An increase of chrome concentration above x = 0.05 reduces the ferromagnetic properties considerably. Analysis of the temperature dependencies of the integral intensity of EPR spectra was carried out using the Curie–Weiss law and the paramagnetic Curie temperature was obtained.

## 1. Introduction

Diluted magnetic semiconductors (DMSs) are of interest, mainly due to the spin–spin exchange interaction between the localized magnetic moments and the band electrons [1,2]. This property of DMS material has potential applications in spin-dependent semiconductor electronics for information processing (spintronics) [3,4,5,6].

A substantial amount of work has been done on Mn-based DMSs. Ferromagnetism has been discovered in materials comparable with the semiconductors used in present electronics, like as (In, Mn)As [7], (Ga, Mn)As [8], Mg [9,10], and Cd_1−x_Mn_x_Te [11,12,13]. Ferromagnetic properties in DMS make them basic component for spintronics. However, their Curie temperatures (T_C_) (e.g., 110 K for (Ga, Mn)As [14]) are not high enough for real applications.

The ferromagnetism in DMS has also been investigated theoretically using a model Hamiltonian [15,16,17]. Dietl et al. proposed Zener’s p–d exchange interaction to describe magnetism [15,16]. This model predicts room-temperature ferromagnetism (RTFM) in (Ga, Mn)N [15,16]. Liu et al. confirmed the ferromagnetic character of the p–d exchange interaction in Cr-based II-VI DMSs [18,19]. Blinowski et al. [20] pointed out very interesting properties of Cr-based DMS and predicted ferromagnetism at room temperature in CdTe:Cr. In the last few years, Cr-doped II-VI DMS compounds have been studied extensively to determine improved ferromagnetic properties and to explore the mechanism behind the half-metallic ferromagnetism (HMF) [21,22,23,24,25,26,27,28].

Some earlier experiments for semiconductor CdTe with Cr using the EPR method were performed. Ludwig and Lorentz [29] studied Cr^1+^ (3d^5^) ions substituted into the cadmium sites in CdTe using electron nuclear double resonance (ENDOR) and EPR. Chromium ions substituting for cadmium ions are expected to be in the Cr^2+^ valence state, i.e., in the 3d^4^ configuration. However, the authors of [29] determined the total spin S = 5/2, g = 1.9997 ± 0.0003, as well the parameters of the cubic zero-field splitting and the hyperfine interaction with second neighbors Cd^111^ and Cd^113^. Vallin and Watkins [30] investigated the EPR spectra of Cr^2+^ in CdTe. The Jahn–Teller coupling coefficients from the changes of the fine structure of the EPR spectra under uniaxial stress were determined. Stefaniuk et al. [31] studied the fine structure of Cr^3+^ ions in CdTe:Cr. The EPR spectra also contained a very broad line assigned to Cr^2+^ ions in CdTe, which are related to magnetic interactions [31,32]. Magnetic properties of Cr-based DMS were studied by Mac et al. [33] and Ko and Blamire [34,35]. Many experiments have been performed to search for DMSs with RTFM and, recently, high T_C_ values have been reported for several systems, e.g., (Ga, Mn)N [36,37,38] (Ga, Cr)N [39], and (ZnCr)Te [40,41,42].

There is a large group of materials with ferromagnetic properties, with a Curie temperature above 500 K (see [43]). However, the goal of this work is to obtain a ferromagnetic semiconductor (DMS) meeting the needs of spintronics.

In this paper, we investigate Cd_1−x_Cr_x_Te powdered bulk crystals that were doped with different concentrations of chrome (up to x = 0.08) during the synthesis process. A wide temperature range of crystals was studied using the EPR method. This study aims to determine the optimal content of Cr ions at which ferromagnetic properties are observed at room temperature.

## 2. Materials and Methods

The crystal preparation procedure consisted of two subsequent stages: the synthesis of polycrystalline source and a crystal growth process, described in more detail in other work [44]. High purity elemental CdTe (99.9999%) supplied by Alfa Aesar and also Cr_2_Te_3_ powder with particle sizes under 325 mesh (99.96%) from Sigma-Aldrich (Saint Louis, MO, USA) were used for (Cd,Cr)Te synthesis. The calculation of the appropriate constituent weights was done considering that chromium atoms substitute cadmium ones and a Cd_1−x_Cr_x_Te solid solution was formed. We studied the pulverized Cd_1−x_Cr_x_Te crystals doped with different nominal concentrations of chrome (up to x = 0.08) during the synthesis process. Growth temperatures were in the range of 1280–1320 °C. The final materials were solid solutions of the source components. The chromium concentrations in the samples were measured by X-ray energy dispersive fluorescent analysis (XFA) (Table 1). All samples for EPR studies were pulverized in an agate mortar. To investigate the crystal structure of samples X-ray diffraction (XRD) was used.

The EPR measurements were performed on an X-band (~9 GHz) spectrometer with a digital registration of the spectra. Part of the EPR measurements was performed on an EPR spectrometer (Bruker multifrequency and multiresonance FT-EPR ELEXSYS E580, Bruker Analytische Messtechnik, Rheinstetten, Germany). The temperature measurements using the digital temperature control system (Bruker ER 4131VT) in the temperature range from 82–381 K were done.

## 3. Results

**Figure 1:**
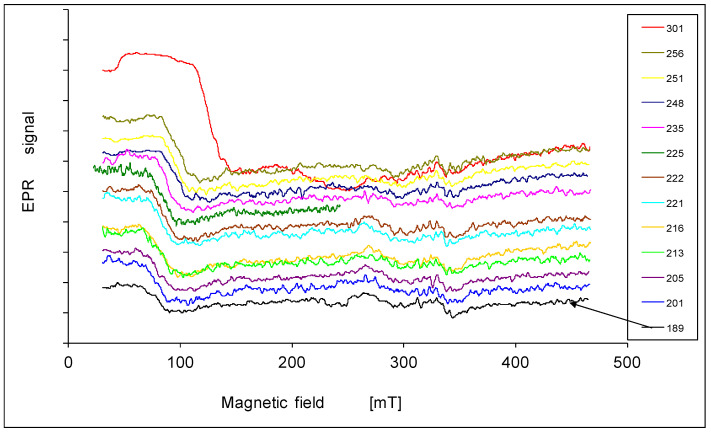
EPR spectra of the sample 1p at various temperatures in K.

**Figure 2:**
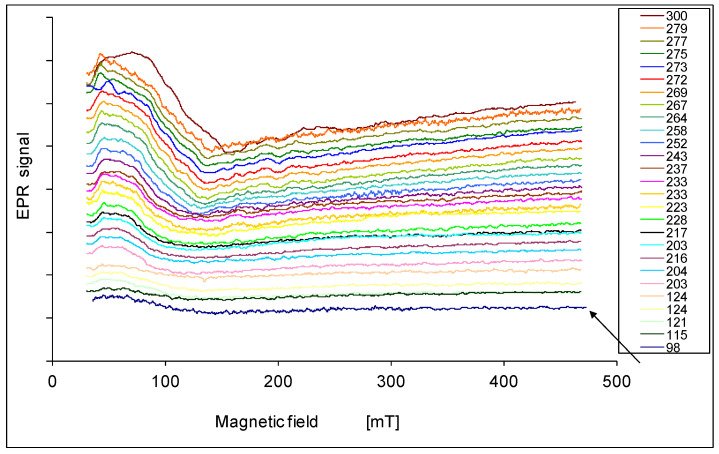
EPR spectra of the sample 2p at various temperatures in K.

**Figure 3:**
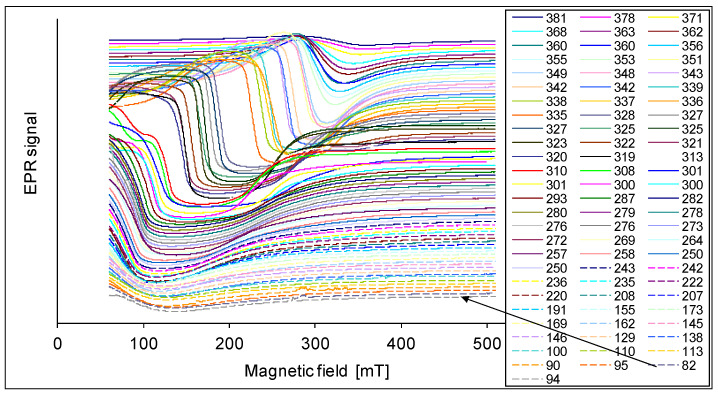
EPR spectra of the sample 3p at various temperatures in K.

**Figure 4:**
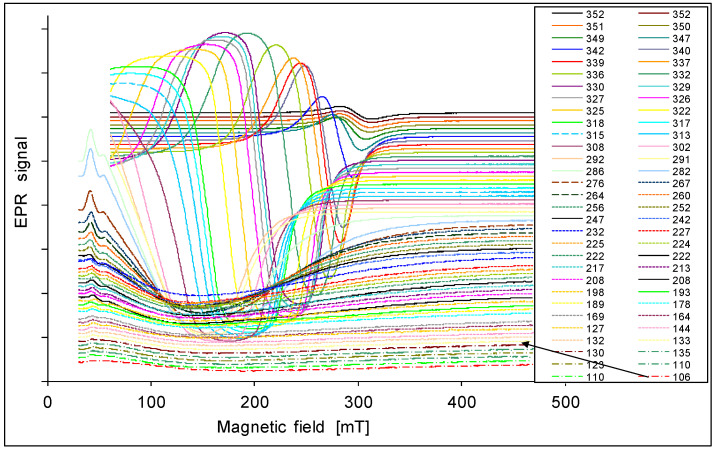
EPR spectra of the sample 4p at various temperatures in K.

**Figure 5:**
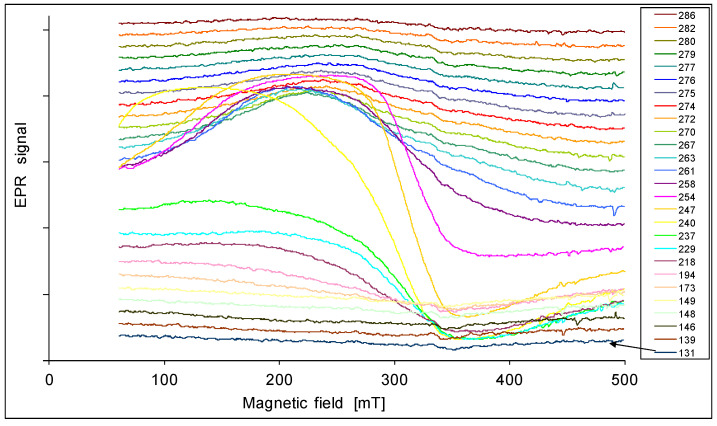
EPR spectra of the sample 5p at various temperatures in K.

The EPR spectra are presented in Figure 1, Figure 2, Figure 3, Figure 4 and Figure 5. In every sample, we observed a wide EPR signal which, however, is irregular for low concentrations of chrome (sample 1p and 2p). In samples 1p and 2p, as well as in sample 5p, we observed a small shift in the EPR line towards a higher magnetic field with increasing temperature. For samples 3p and 4p, very regular shapes of the EPR spectra above room temperature (Figure 3 and Figure 4) were obtained. At lower temperatures, the resonance field shifted towards lower magnetic fields, and then only a part of the EPR line was observed. The EPR lines presented in Figure 1, Figure 2, Figure 3, Figure 4 and Figure 5 are arranged such that they are shifted upwards with increasing temperature, as indicated by the legend. The amplitudes of the EPR signals for samples 1p, 2p, and 3p became smaller when the temperature was lowered from room temperature. However, for samples 4p and 5p, we first observed an increase in the amplitude of the signal with reduced temperature, but below a certain temperature a decrease was noted. Furthermore, the linewidth strongly depended on both, the temperature and the sample, i.e., the concentration of chromium.

## 4. Discussion

The ferromagnetic properties of semiconductors are the subject of wide investigations at present. It is known that for similar materials, the shape of an EPR line is closely related to the kind of magnetic interaction [45,46,47,48,49]. Since the exact mechanisms of ferromagnetism in the present material are still not known, the choice of the theoretical curve for the fitting of the experimental data is not straightforward. Therefore, in the initial stage of the research, we focused on the determination of the appropriate concentration of chromium, depending on strong ferromagnetic properties at high temperatures. This was realized by measurements of the temperature dependences of the peak-to-peak linewidth H_pp_, the peak amplitude A, and the resonance field H_r_. The latter is in simple relation to the effective gyromagnetic factor g_e_. Since the majority of the measured EPR spectra are not complete due to the large linewidth and their shifts towards smaller magnetic fields for lower temperatures, it is impossible to get the line parameters directly from the spectra. Thus, these parameters were determined by fitting the resonance line to the theoretical curve by using the standard computer program OriginPro 7.5, OriginLab, Northampton, MA, USA. For numerical analysis, a Lorentzian function was applied [50],

(1) $$y=P-\frac{3^{\frac{1}{2}}\times H_{\mathrm{pp}}\times \left(\chi -H_{r}\right)}{\pi \times {\left(0.75\times H_{\mathrm{pp}}^{2}+{\left(\chi -H_{r}\right)}^{2}\right)}^{2}}\times \;A$$

where P is a constant, H_pp_ is the peak-to-peak-width, H_r_ is the resonance field, and A is the peak amplitude. The analysis of the shape of the experimental line shows its complex structure. In this work, we used fitting with a single Lorentz shape line as the closest match to the real one. We are aware that some parameters are different (e.g., intensity of line A), but, at the same time, other parameters are obtained with good accuracy (e.g., resonance field H_r_).

**Figure 6:**
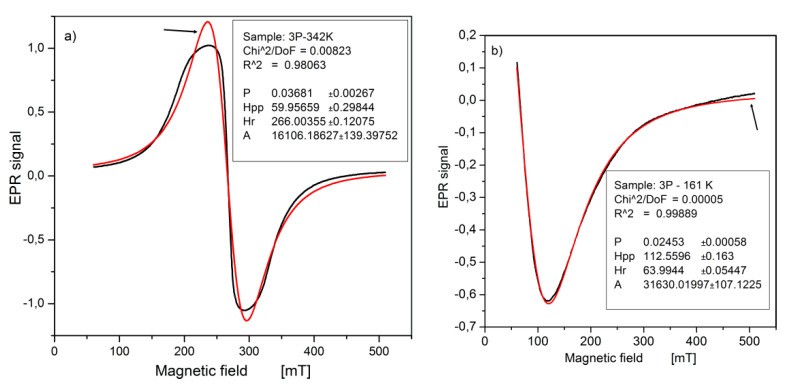
Representative EPR spectra of Cd_1__−x_Cr_x_Te powdered crystals: (**a**) sample No. 3p at T = 342 K, (**b**) sample No 3p at T = 161 K. The arrow marks the theoretical line. The fitting parameters are given in the legend.

As an example, in Figure 6, we show the result of the fitting of an experimental spectrum to a Lorentzian curve. As it is shown in Figure 6b for the incomplete EPR lines very well-fitting results were received. However, the fitting to the whole or considerable parts of the visible lines as one can see in Figure 6a was worse. For example, the accuracy of the fitting for a small number of some very irregular lines, the EPR spectrum of the sample 5p at the temperature around 240 K, was poor. The value of the resonance field H_r_ is very well reproduced, whereas the linewidth H_pp_ shows deviations up to maximal errors of several percent. However, we are interested in the temperature dependence of H_pp_. From such diagrams, we are able with good accuracy to find the temperature in which the large changes of H_pp_ occur.

**Figure 7:**
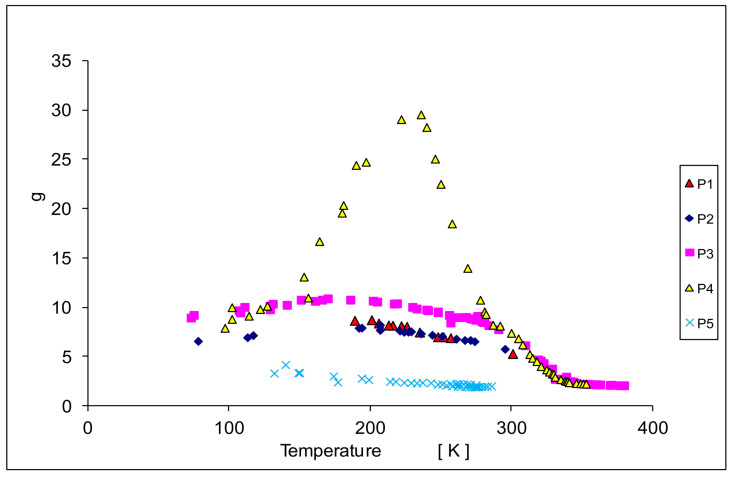
The temperature dependence of the effective gyromagnetic factor g_e_.

**Figure 8:**
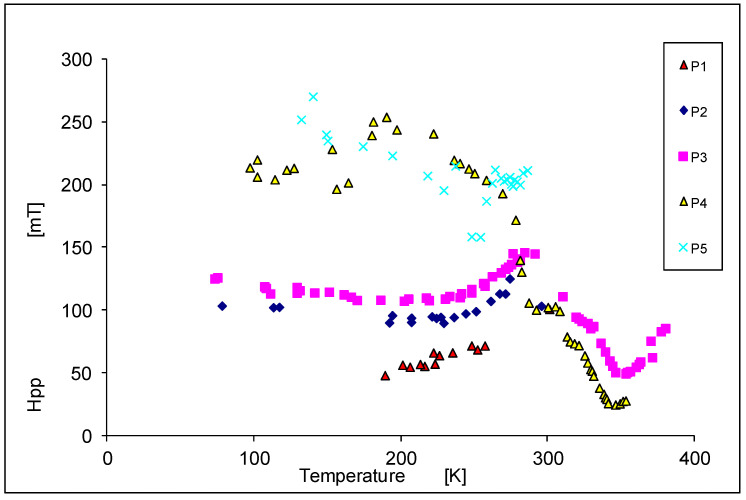
The temperature dependence of the peak-to-peak linewidth (H_pp_).

**Figure 9:**
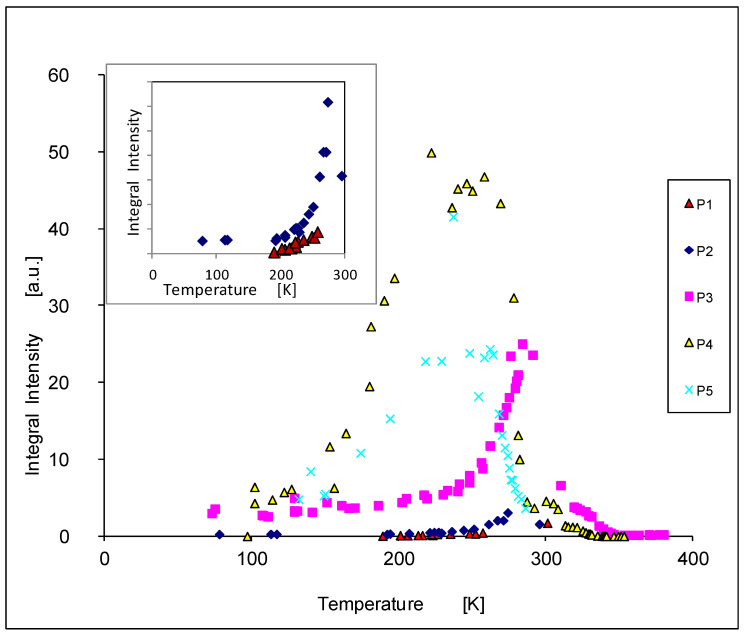
The temperature dependence of the integral intensity I_t_ ($I_{t}=A\;\times {\;H}_{\mathrm{pp}}^{2}$).

Determining parameters of the EPR spectra such as H_r_ (or g_e_), H_pp_, and A, are presented in Figure 7, Figure 8 and Figure 9. In Figure 7 the temperature dependence of the effective gyromagnetic factor g_e_ is shown for all samples. In a paramagnetic material, the applied external Zeeman field is equal to the local field and the g-factor is obtained from the Zeeman formula. The effect is more complicated in ferromagnets. The local resonance field is a superposition of the external field H_0_, the demagnetizing field, and other contributions to the magnetic anisotropy energy [49,51]. We observe a paramagnetic phase at higher temperatures with g_e_ close to 2. However, at lower temperatures, the value of g_e_ increases due to intrinsic magnetic fields responsible for ferromagnetism. A larger value of g_e_ is related to a larger intrinsic magnetic field. The samples 1p and 2p with small concentrations of chrome show ferromagnetic properties with g_e_ about 8. With the increase of the concentration of chrome as in sample 3p, an increase of g_e_ (g_e_ = 10) was observed. In sample 4p it accepts a very large value close to g_e_ = 30. This is a very high value in comparison with other materials. Further increase of concentration like in the sample 5p, an interesting effect is seen, that g_e_ again accepts the value of about 2. A possible explanation may be the appearance of antiferromagnetic interactions. Similar results have been found for Mn in CaMn_1−x_Ru_x_O_3_ [47].

In Figure 8, we show the temperature dependence of linewidth. For the samples 1p, 2p, and 3p, we find a maximum for H_pp_ approximately at room temperature. For sample 4p the maximum is found close to 170 K. However, for sample 5p the linewidth increases with lower temperatures. In the samples 2p and 3p, the linewidth remains almost constant below 160 K. For the samples 3p and 4p we observe a decrease of the linewidth when the temperature is raised above room temperature, and we get a minimum for H_pp_ in the range 340–350 K. For higher temperatures we find an increase of the linewidth again and simultaneously a reduction of the intensity; this is the typical behavior of a paramagnetic sample [49]. The largest change of the linewidth H_pp_ in dependence of the temperature is in the sample 4p: it changes from 25 mT at 346 K to 250 mT at 180 K.

The temperature dependence of the integral intensity is shown in Figure 9. We see that for the samples with higher concentrations of chrome the maxima of the intensities are shifted to lower temperatures. Furthermore, we observe that the intensities rise with increasing concentrations of chrome (samples 1p, 2p 3p); the maximum is obtained for the sample 4p, and then it decreases again like in sample 5p.

**Figure 10:**
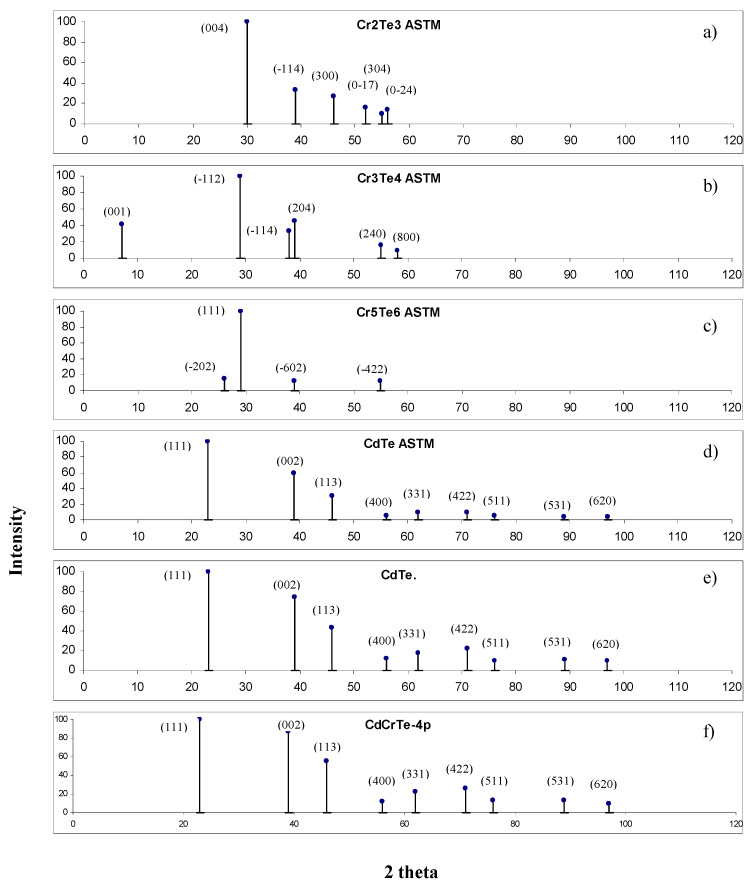
XRD spectra of CrTe, CdTe, (**a**–**d**)—according to the ASTM tables, and CdTe, CdCrTe (**e**,**f**)—experimental results.

The presented results were completed by studies of the chemical and crystallographic homogeneity of the samples. For this aim, we performed XRD measurements. The spectra of our samples were compared with those of pure CdTe as well as with CrTe phases such as Cr_7_Te_8_, Cr_3_Te_4_, Cr_5_Te_6_, etc. which are the most likely inclusions of the second phase (Cr_2_Te_3_ was an initial component in the process of the CdCrTe synthesis). In Figure 10 spectra with those given by standard ASTM X-ray powder data were compared. The main diffraction peaks of our sample no. 4p (Figure 10f) corresponds very well with the zinc-blende structure of the CdTe crystal (Figure 10d,e). We have not detected diffraction peaks from CrTe phases which crystallize in a different structure—in the structure of NiAs [52] (see Figure 10a–c).

**Figure 11:**
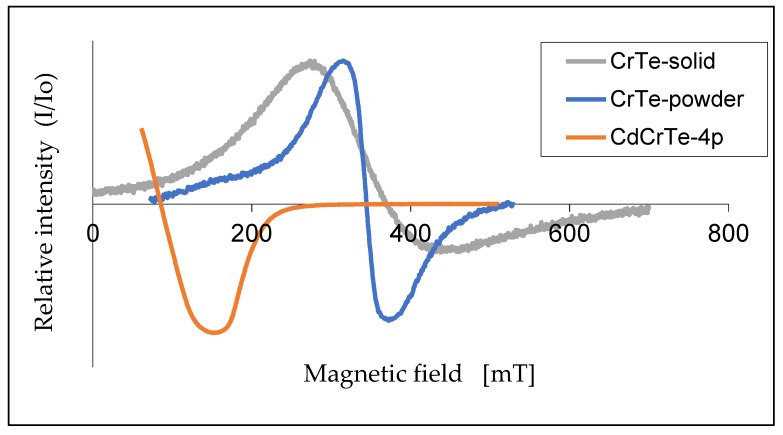
EPR spectra of powered, solid Cr_2_Te_3_, and the sample 4p (T≈260 K).

The lack of the second phase in our samples is also demonstrated by the EPR measurements of the Cr_2_Te_3_ powder (the initial material for CdCrTe synthesis), as well as of the Cr_2_Te_3_ solid (premelted in the conditions of CdCrTe synthesis). These spectra for the temperature of 260 K are shown in Figure 11. They also differ considerably from those obtained for CdCrTe. The resonance fields are 341 mT and 347 mT for powdered and solid Cr_2_Te_3_, respectively, whereas for CdCrTe it has a value of 95 mT. The EPR line of Cr_2_Te_3_ at room temperature (paramagnetic phase) is also of Lorentzian shape, however, with decreasing temperature the shape changes towards a Dysonian function, particularly at low temperatures. The positions of the lines (of Cr_2_Te_3_ powdered and solid) do not change with temperature contrary to the EPR line of CdCrTe.

**Figure 12:**
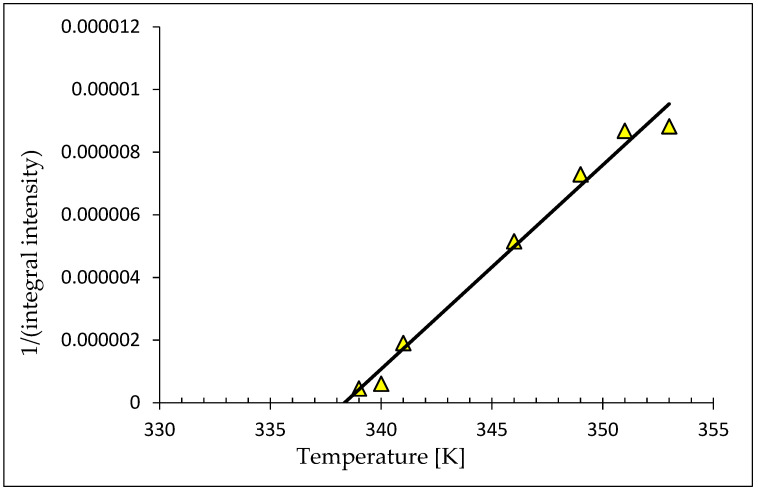
Temperature dependence of the 1/(integral intensity) of the sample 4p.

We used the Curie–Weiss law to analyze the temperature dependences of the integral intensity, which is directly proportional to the magnetic susceptibility χ. A linear increase of χ^−1^ (T) at higher temperatures can be fitted to the Curie-Weiss law [53,54],

(χ − χ_0_)^−1^(T) = (T − θ_cw_)/C, (2)

where C is the Curie constant, θ_cw_ is the paramagnetic Curie temperature, and χ_0_ is a temperature-independent term to account for the diamagnetic host and any Pauli paramagnetism contribution. Figure 12 displays the temperature dependence of the quantity (χ − χ_0_)^−1^ for sample 4p. The lines are linear extrapolations illustrating the ferromagnetic (positive) Curie–Weiss temperatures. Fitting yields the following values: for sample 4p-θ(x) = 338.3 K and C(x) = 5.91 × 10^−7^; for sample 3p-θ(x) = 333 K and C(x) = 7.51 × 10^−6^; for sample 2p-θ(x) = 250 K and C(x) = 6.25 × 10^−9^. The Curie constant has a dimension of $\frac{{\mathrm{Km}}^{3}}{\mathrm{kg}}$.

Ko et al. [34,35] reported a Curie temperature for a bulk Cr-doped CdTe crystal of T_C_ = 354 K for x = 0.06, which agrees well with our observations (Figure 7, Figure 8 and Figure 9) and Curie temperature T_C_ = 338.3 K for sample 4pg_e_ values for the samples 3p, 4p, and 5p show small differences in the region of higher temperatures (paramagnetic region). A similar effect was observed by Son et al. [46] for Mn in Cd_1−x_Mn_x_Te.

A very large g_e_ factor (up to 30) observed for the 4p sample, shows a very strong intrinsic magnetic field, which, together with a high Curie temperature may have its origin in a superexchange interaction of Cr ions in the Cr-dissolved CdTe phase. The observed effects may indicate superparamagnetism [55,56,57].

## 5. Conclusions

The fine structure of the spectra only in the sample with a low concentration of Cr atoms (sample 1p, Figure 1) is visible. It is superimposed on the one intensive line positioned at a lower magnetic field.

In the samples with a concentration of Cr from x = 0.002 to 0.08, a broad line in the EPR spectrum is observed. The shape of this line is symmetric and regular for samples 3p and 4p. The position of the line strongly depends on the temperature.

At several characteristic temperatures, a change of the slope of the temperature depends on the resonance field (H_r_), the linewidth (H_pp_), and the total intensity (I_t_) was observed. At about 160 K this is seen for H_pp_, I_t_, and especially for the g_e_ factor to the different range and depending on the Cr concentration; at 240 K for the g_e_(T) dependence especially in sample 4p; at 280 K (room temperature) for H_pp_(T) in all samples, and to a smaller extent for g_e_(T) and I_t_(T); in the temperature range 340–350 K for H_pp_, g_e_, and I_t_ in the samples 3p and 4p.

g_e_ values for the samples 3p, 4p, and 5p show small differences in the region of higher temperatures (paramagnetic region). The value of g_e_ is higher for larger concentrations of Mn. However, we obtained the values 2.18, 2.28, and 1.93 for the samples 3p, 4p, and 5p, respectively. Thus, g_e_ does not shift to higher values with increasing concentration of Cr but achieves a maximum for x = 0.05 (sample 4p).

The most interesting magnetic properties for the sample 4p in the vicinity of room temperature, for all EPR line parameters, were observed. A very large g_e_ factor (up to 30) shows a very strong intrinsic magnetic field. This intrinsic ferromagnetism together with a high Curie temperature may have its origin in a superexchange interaction of Cr ions in the Cr-dissolved CdTe phase. The observed effects indicate possible superparamagnetism. Detailed research for this sample will be conducted further, especially taking into account the precise analysis of the line shape, as well as the registration of the spectrum in the negative field. The use of a simple method of sintered powdered materials: Cd, Te, and Cr_2_Te_3_ allows for easy control of the content of chromium ions in Cd_1−x_Cr_x_Te. The DMS obtained in this way shows ferromagnetic properties at room temperature, especially in the range x = 0.05–0.1.

It is found that an increase of the concentration of Cr up to x = 0.05 enlarges the ferromagnetic properties, but a further augmentation of the Cr concentration reduces ferromagnetism and may lead to the presence of antiferromagnetism.

## Figures and Tables

**Table 1:** Samples investigated.

Sample	Growth Temperature, °C	The Concentration of Cr in Cd_1−x_Cr_x_Te	
		Charge, x	XFA Measurement, x
1p	1280	0.04	0.002
2p	1300	0.01	0.008
3p	1300–1310	0.05	0.04
4p	1320	0.05	0.05
5p	1320	0.1	0.08

## Data Availability

Data sharing is not applicable to this article.
